# Application of a Conceptual Model for Translational Science Impact

**DOI:** 10.1017/cts.2025.10206

**Published:** 2025-12-03

**Authors:** Jessica H. Presley, Shani Worrell, Alexandria Jauregui-Dusseau, Christi A. Madden, Laura P. James

**Affiliations:** The Translational Research Institute, University of Arkansas for Medical Sciences, Little Rock, AR, USA

**Keywords:** Translational science, TSBM, impact evaluation, CTSA, translational science Principles

## Introduction

The latest National Center for Advancing Translational Sciences (NCATS) Clinical and Translational Science Award (CTSA) Funding Opportunity Announcements (FOA) emphasized translational science (TS) activities that build an environment to support efficient and effective TS and research [1]. Past CTSA FOAs did not explicitly distinguish between TS activities and translational research (TR) activities. TS and TR are closely related, but distinct. “Translational research” traverses a particular step of the translational process for a particular target or disease. Conversely, “translational science” seeks to understand the scientific and operational principles underlying each step of the TR process [2], so that generalizable knowledge and approaches that accelerate TR can be tested, refined, and shared with the research community [3]. The CTSA community grappled with the new TS focus and was challenged with the distinction between TS and TR [4–6]. Further, evaluators and program administrators struggled with articulating a clear pathway between TS activities and TS impacts.

TS principles were introduced by NCATS in 2022 [3] and were stressed in CTSA FOAs. The TS principles describe effective approaches to TS, though many of the principles can be applied to effective TR as well. Administrators across the CTSA consortium have been exploring a variety of approaches to shift hubs and programs from TR to TS. Schneider et al. explored the use of the TS principles to distinguish between TR and TS within pilot studies and determined the extent of alignment with NCAT’s vision of TS [4]. In a paper published by Kim et al., the authors described the challenges the Einstein-Montefiore CTSA hub encountered as the institution set out to promote TS research [5]. Kim et al. called for systematic examination of how the CTSA community should pursue TS. In Glauber et al.’s paper, they described a virtual retreat for personnel at the Duke CTSA hub that promoted organizational learning about TS and discussion of challenges and opportunities [6]. While there is still ongoing discussion of how to effectively shift to TS, there is greater consensus around approaches to measuring CTSA hub impact.

The Translational Sciences Benefits Model (TSBM) is a framework that has been widely adopted by the CTSA evaluation community to assess translational impacts [7]. The TSBM was originally developed with a focus on health and social benefits resulting from TR. While TR activities have direct links to TSBM constructs, TS activities are often indirectly linked to the 30 health and social benefits identified by the model. Essentially, TS directly impacts the translational process and there is a gap in understanding how impacting the translational process influences health and social benefits. There have been previous efforts to adapt the TSBM to incorporate additional domains. Most recently, La Manna et al. sought to incorporate equity subdomains with associated benefits within each TSBM domain [8]. In another recent study, Emmons et al. extended the TSBM with the addition of an implementation science outcomes domain as a precursor to and influencer of health and social benefits [9]. Although the incorporation of implementation science expands the TSBM to accommodate an important TS endeavor within the dissemination and implementation stage, to our knowledge, there has been no effort to model how discrete TS activities connect to health and social benefits.

The Translational Research Institute (TRI) at the University of Arkansas for Medical Sciences was refunded in 2024 under a CTSA FOA that contained the new TS emphasis. To redefine evaluation under this new FOA, the TRI developed a conceptual model to elucidate how TS activities increase the efficiency and effectiveness of TR and lead to TS health and social impacts.

## Materials and methods

Evaluations of CTSA institutions are highly complex, assessing an array of activities and resources across workforce development, community engagement, pilot funding, hub resources and services, informatics, and research. CTSA evaluation demands intimate knowledge of the programs that operate within strategy areas to connect activities to hub objectives and outcomes. Conceptual frameworks are useful tools for engaging stakeholders in articulating program theory and driving evaluation [10]. Therefore, the creation of a conceptual framework for TS impact was undertaken to guide program and evaluation planning within the TRI.

Informed by literature on TS principles and the TSBM, an initial draft of a conceptual framework to address this gap was developed in August 2022 and underwent multiple iterations in collaboration with TRI leadership over an 18-month period. Additional revisions were informed by feedback from CTSA evaluators through a poster presentation at the American Evaluation Association Annual Meeting in 2024 and by CTSA principal investigators through three presentations at cross-hub meetings from 2023 through 2024. The framework was received enthusiastically across presentations. Feedback reinforced a broad need for deeper understanding of the concepts included in the framework and informed clarification of the relationship between TS and TR.

## Results

**Figure 1.**
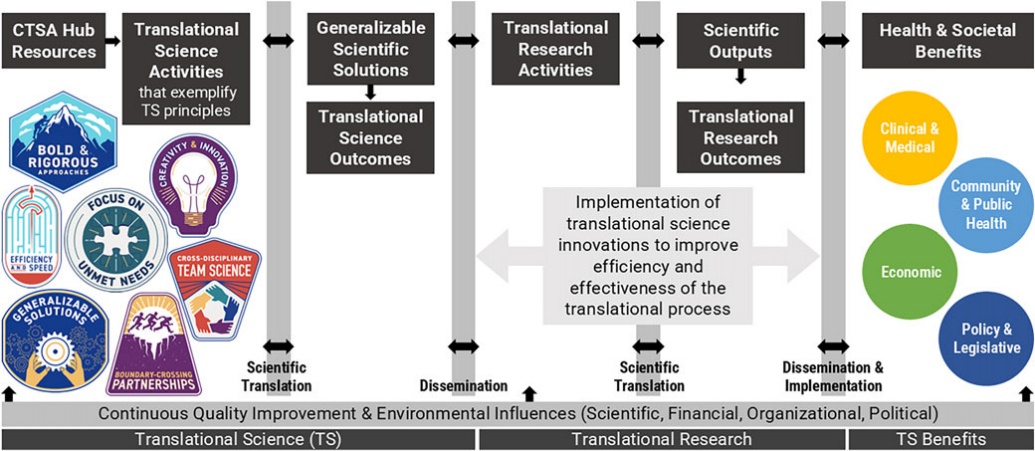
TRI’s Conceptual Model for Translational Science Impact. Adapted from the TSBM and Translating for Impact Toolkit © 2017–2023 created by the Institute of Clinical and Translational Sciences at Washington University in St Louis and available at translationalsciencebenefitsmodel.wustl.edu, licensed under CC BY-NC-SA 4.0 [11]. The TS principles are made available for use by NCATS via a CC BY 4.0 License [12].

The resulting Conceptual Model for Translational Science Impact integrates the TS principles and the TSBM. The model shown in Figure 1 explicates a pathway from TS activities that exemplify TS principles to TSBM benefits.

CTSA FOAs emphasize TS activities, which is the scope of investigation bound by the “Translational Science” bar at the bottom of the model. The overall aim of NCATS’ CTSA program is to support institutions in building the capacity and environment for high quality clinical and TS, with the goal of achieving large health and social impacts. Therefore, the majority of TRI’s CTSA hub activities are aimed at providing resources and supports to investigators, (“CTSA Hub Resources” in Figure 1) and in creating an environment where TS principles can be actualized. Investigators, therefore, are able to conduct TS activities that exemplify the TS principles and lead to the development of TS innovations that are generalizable scientific solutions, as shown in Figure 1. These generalizable solutions are then disseminated and embedded into TR activities, enhancing the translational process (as illustrated by the “Translational Research” bar at the bottom of the model). Implementation of the TS innovation strengthens the efficacy of TR which is likely to lead to significant health and social benefits or impact. For this reason, the model presents the TSBM as a distal outcome (Health & Social Benefits” on the right side of Figure 1) of TS activities.

The linear presentation of the model is useful for mapping downstream impacts of TS. A limitation of this model, however, is the oversimplification of the model to show TS and TR as two distinct and linear processes. The NCATS Translational Spectrum describes TS as a circular process where new approaches are developed, their usefulness demonstrated, and findings disseminated [13]. TS encompasses the TR process and benefits from a reciprocal relationship, creating feedback loops between TS and TR. The model attempts to illustrate these relationships with bidirectional arrows.

**Figure 2.**
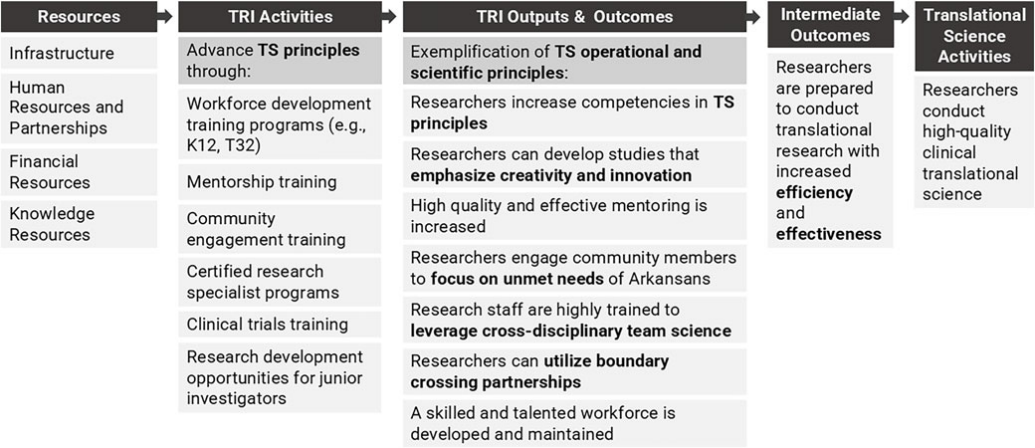
TRI’s logic model for workforce development.

The TRI’s evaluation team has adopted the Conceptual Model for Translational Science Impact to elucidate the pathways through which TRI activities influence health and social benefits for rural populations in Arkansas. TRI logic models can be appended to the conceptual model to map how hub activities support TS innovations that can be adopted and implemented by researchers to influence health and social benefits. Considering hub outputs and outcomes through the lens of TS principles helps evaluators measure how the principles are bolstered by the institution to build an environment to support efficient and effective TS. For example, the logic model in Figure 2 shows how equipping trainees with competencies in TS principles prepares them to conduct high quality clinical TS. The logic model and conceptual model can be linked at “Translational Science Activities,” which appears on both the logic model as an intermediate to long-term outcome of workforce development activities and on the conceptual model as the initial activity to which everything is mapped. When linked, the activities, outputs, and outcomes of the logic model explicate “Hub Resources” that support “Translational Science Activities” in the conceptual model.

To illustrate use of the logic model and conceptual model, consider the following example. The TRI’s K12 program supports the career development of a trainee through programing, mentorship, training, and support (see “TRI Activities” in the logic model in Figure 2). As a result, the trainee experiences growth in TS competencies and the ability to apply TS principles (see “TRI Outcomes” in Figure 2). These new competencies and skills allow the trainee to conduct an efficient and effective hub-funded research project with a TR aim of improving the diagnosis of a specific condition among neonate patients and a TS aim of improving the consent process for this vulnerable population to meet study enrollment goals (see “Translational Science Activities” and “Translational Research Activities” in the conceptual model in Figure 1). The trainee’s project leads to the development of a TS innovation, a new approach to the consent process, that provides a generalizable and scientific solution to meet enrollment goals where many studies fall short. The innovation ensures a representative sample is achieved, thereby increasing the efficiency and efficacy of the trainee’s TR study and increasing the likelihood of a significant breakthrough in the diagnosis of a medical condition among neonates and achieving health and social benefits. Additionally, the new approach to consenting a vulnerable population is generalizable to other TR studies. These studies adopt and implement the innovation to improve the efficiency and effectiveness of the studies by meeting participant enrollment goals, which leads to further health and social benefits across research projects. Output and outcome metrics can be tracked along each phase, including at the hub- and trainee-levels in the logic model in Figure 2 and at the project-level tracking TS and TR hub supported projects from implementation to health and social impacts using the conceptual model in Figure 1. TS and TR feedback loops are illustrated in the model when additional roadblocks to the translational process are identified that inspire new TS studies to develop novel TS innovations that will improve TR. For example, during the dissemination and implementation phase, the need for an improved strategy for adoption and implementation of the new diagnostic method for neonates could inform another TS study.

## Discussion

The TRI’s Conceptual Model for Translational Science Impact maps the journey from the development of a TS innovation through adoption and integration of the innovation within TR studies to produce more effective TR with greater health and social benefits. Better understanding of outputs and outcomes of TS has assisted the TRI’s evaluation team in identifying potential metrics to measure TS innovations. Metrics go beyond traditional counts of publications, grants, and patents to assessing adoption and implementation of innovations in research studies, efficiency and efficacy of the study as a result of adoption of the innovation, and assessment of how the innovation has enhanced associated health and social benefits of TR studies. Systems to assess adoption and implementation are not yet in place at the TRI. The development of a system to track the adoption of TS innovations is a potential area of collaboration for CTSA evaluators that could take cues from implementation science. Development of a classification system for types of translational solutions (e.g., participant recruitment, predictive efficacy, etc.) along with associated outcomes and aligned metrics could be a useful tool for assessing effectiveness of TS innovations. Future research might consider whether particular health and social benefits are more often associated with TS innovations than others.

TRI is using the model to develop explanatory logic models to guide impact evaluation for modules and programs. As part of testing this model, TRI is now studying measurement of TRI activities in terms of the TS principles. Approaches to measure TS principles are in development and may include assessment of changes in TS competencies among trainees and a measure of the application of TS principles in hub supported trainee research.

The Conceptual Model for Translational Science Impact has been beneficial for TRI evaluators to articulate how TS activities guided by TS principles result in TS innovations that bolster the efficiency and effectiveness of TR, as emphasized by FOAs from NCATS, and connect TS to the TSBM’s health and social benefits. This conceptual framework can be used by other CTSA hubs to identify and emphasize TS focused activities and map new pathways to health and social impact.
